# An unusual case of unilateral sinus disease may reveal the presence of a retained foreign body

**DOI:** 10.1016/j.ijscr.2020.10.074

**Published:** 2020-10-22

**Authors:** Matteo Gelardi, Nicola De Candia, Eleonora M.C. Trecca, Michele Cassano, Nicola A.A. Quaranta

**Affiliations:** a University Hospital of Foggia, Department of Otolaryngology- Head and Neck Surgery, Foggia, Italy; b University of Bari ‘Aldo Moro’, Department of Otolaryngology- Head and Neck Surgery, Bari, Italy

**Keywords:** Foreign bodies, Sinus surgery, FESS, Rhinology, Sinonasal diseases, Case report

## Abstract

**Introduction:**

Differential diagnosis of unilateral sinus disease (USD) is important in clinical practice as it can be broad, and for which timely and effective treatment is important. With this report we would like to present a case of a patient with a foreign body retained in the left maxillary sinus for eight years.

**Presentation of case:**

A 50-year-old gardener referred to have been injured on the job in 2010. Even though he was wearing his safety helmet with face shield and earmuffs, he had the sensation to have been hit by an object coming from the soil, while he was using his grass trimmer. However, the patient was asymptomatic with no complications. After eight years, the man started experiencing left nasal obstruction associated with purulent discharge and ipsilateral headache. A fetid odor coming from inside the nose and described as “the carcass of a dog” led him to seek medical attention. The patient underwent a functional endoscopic sinus surgery (FESS) with septoplasty and a foreign body, consisting of a metal nail, was retrieved.

**Discussion:**

This case highlights a two key learning points. The first one concerns occupational safety measures; full-face shield helmets are the best option and should be employed in order to avoid the penetration of any possibly dangerous materials. Secondly, grass trimmers with more sophisticated designs are required in order to prevent injuries like this.

**Conclusions:**

Besides being peculiar because of the onset of clinical symptoms, this case report highlights the importance of full-face shield helmets and certified machinery.

## Highlights

• Unilateral sinus disease (USD) can be due to a wide range of conditions.
• This report presents the case of a patient with a foreign body retained in the left maxillary sinus.
• The patient underwent a functional endoscopic sinus surgery (FESS) with septoplasty.
• Besides being peculiar for the onset of clinical symptoms, this case highlights the importance of occupational safety measures to prevent foreign body aspiration.

## 1 Introduction

The differential diagnosis of unilateral sinus disease (USD) is important in the clinical practice as it can be due to a wide range of conditions, such as periodontal diseases, tumors, fungus balls and retained foreign bodies, whose timely and effective treatment is important [1]. However, the presence of foreign bodies often can be silent for many years and this sometimes leads to a misdiagnosis or delayed treatment. The maxillary sinus is the most commonly affected sinus, involving 69 % and 95 % of cases in unilateral and bilateral sinus disease respectively. Nonetheless, patients with USD more commonly present with acute rhinosinusitis than patients with bilateral sinus disease. This should be taken into consideration in the workup and management of patients with USD [2].

Performing a literature review, the most frequent causes of USD appear to be chronic rhinosinusitis (CRS), followed by mycosis, inverted papilloma and cancer, in 1–3 % of cases [3]. Another common but underreported cause of maxillary sinusitis is odontogenic with a variable incidence ranging from the 10 to 12 % [4] to the 40 % [5,6]; related to the diffusion of an odontogenic infection involving more often the posterior maxillary dentition (i.e. molars, premolars), as well as to oral surgical procedures and maxillary dental trauma [7]. Additionally, the presence of displaced dental foreign bodies well represented in the literature with several case reports should be acknowledged and investigated while taking the patient’s history and with radiological examinations (i.e. dental X-ray, CT scan) [4,[8], [9], [10]]. However, a recent article commented that the awareness of this condition remains poor, especially among young Otolaryngologists and their trainees [11]. Even lower on the differential diagnosis of USD is the presence of foreign bodies of non-odontogenic nature. Given this prior literature, we would like to present the case of a patient with a non-odontogenic foreign body retained in the maxillary sinus for eight years, which represents one of the longest times of retainment accompanied by a subclinical pattern, to the best of our knowledge. The presentation of this case is compliant with the SCARE guidelines 2018 [12].

## 2 Case report

**Fig. 1:**
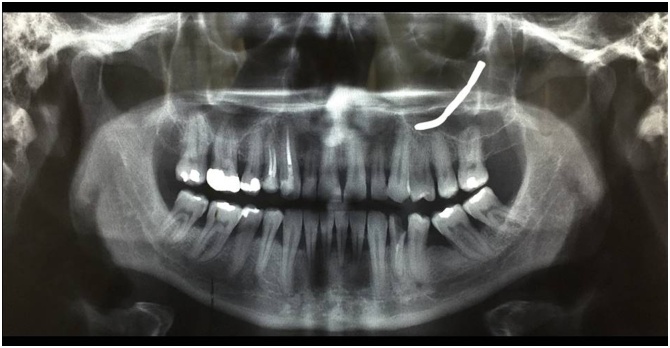
The dental X-ray evidenced the “presence of a metal prosthesis located in the left side of the facial skeleton and the absence of areas of apical/radicular osteolysis affecting the teeth”.

**Fig. 2:**
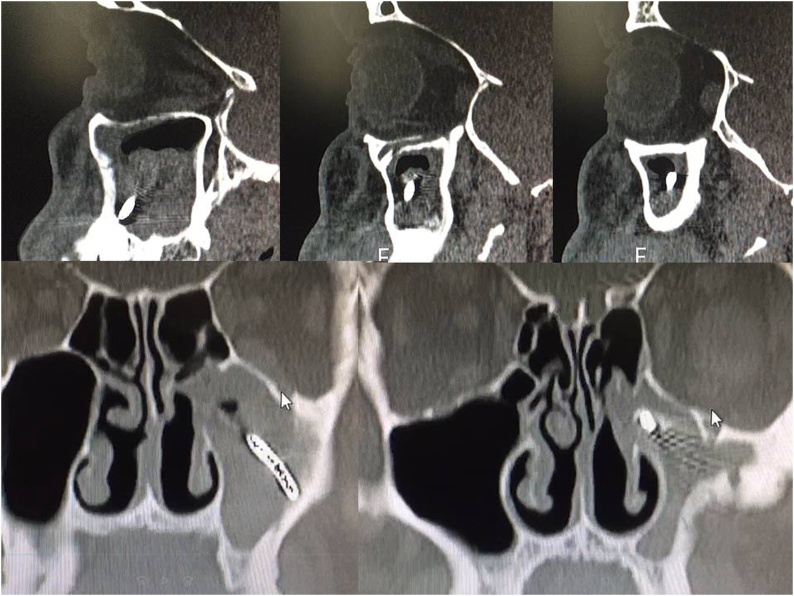
Sinus computed tomography (CT) executed without contrast evidenced the “presence of a metal foreign body with thread-like shape located in the region of the left maxillary sinus, which determined intense inflammatory reaction of the sinonasal mucosa interesting the same maxillary sinus”. Additionally, “the ipsilateral anterior ethmoidal air cells were partially involved”. Radiological findings of minor importance were the presence of left concha bullosa and nasal septum deviation, while the other nasal sinuses were within normal limits.

A 50-year-old man in good health with no prior medical history, who has been working as a gardener for more than 30 years, referred to have been injured on the job in May 2010. He explained that he experienced a violent blow to the left zygomatic region, while he was using his grass trimmer (Professional Grass Trimmer, model TR 600 by Meccanica Benassi ®-Italy). Even though he was wearing his safety helmet with face shield and earmuffs, he had the sensation to have been hit by an object coming from the soil. After the trauma, the patient immediately noticed a 2-mm lesion on his skin; he thought that it was insignificant so much so that he did not seek care (i.e. hemostasis, suturing and wound management). A few hours later the man suffered from burning pain and ipsilateral rhinorrhea with mild bleeding; however, the patient did not feel the presence of any foreign bodies in the subcutaneous tissue on palpation. Additionally, the zygomatic region did not present edema or bruising. In September 2010, the patient visited his Dentist for a regular dental check-up. As shown in Fig. 1, the dental X-ray revealed the “presence of a metal prosthesis located in the left side of the facial skeleton and the absence of areas of apical/radicular osteolysis affecting the teeth”. Surprisingly, the patient had never undergone any dental implant operations from the time of injury to his presentation to our clinic. Given these last findings the dentist suggested the man to consult an Otolaryngologist, but the patient decided to follow this suggestion only after five years, when he started experiencing facial pain. The fiberoptic nasolaryngological examination was within normal limits, except for a deviation of the nasal septum. The computed tomography (CT) of the paranasal sinuses executed without contrast evidenced the “presence of a metal foreign body with thread-like shape located in the region of the left maxillary sinus, which determined intense inflammatory reaction of the sinonasal mucosa interesting the same maxillary sinus”. Additionally, “the ipsilateral anterior ethmoidal air cells were partially involved”. Radiological findings of minor importance were the presence of left concha bullosa and nasal septum deviation, while the other paranasal sinuses were within normal limits (Fig. 2). Given this radiology report, the patient underwent Functional Endoscopic Sinus Surgery (FESS) with septoplasty in an Otolaryngology (ORL) department of a different hospital on January 28th, 2015. The pre-operative testing, including blood chemistry test, electrocardiogram and chest X-ray, were all within normal limits. General endotracheal anesthesia was induced, but unfortunately the Otolaryngologist was not able to remove the foreign body from the maxillary sinus. However, the patient was ensured that the foreign body would not cause him any additional symptoms or harm and he was discharged on post-operative day 3. Post-surgical care at home included nasal irrigations, topical antibiotics, steroids and proton pump inhibitors. In addition, the first follow-up visit was scheduled at 15 days after the surgery in order to remove silastic sheeting. Three years later in 2018, the patient started experiencing left nasal obstruction accompanied by purulent blackish discharge and ipsilateral headache. His chief complaint however was a fetid odor coming from inside the nose and described it as “the carcass of a dog”. The sensation generally worsened in the morning, especially when he leant forward. For this reason, the patient presented to the department of ORL of Bari University Hospital.

### 2.1 Ear, Nose and Throat (ENT) examination

The examination of the face did not reveal any cutaneous abnormalities; such as skin pigmentation or abnormal texture of the left zygomatic region or any other areas. Diagnostic nasal endoscopy evidenced the outcomes of the previous septoplasty with a little perforation on the anterior nasal septum and a purulent discharge in the middle meatus. No nasal polyps were evidenced and the rhinopharynx was normal except for the presence of the nasal discharge. The oropharynx examination was mostly normal with normotrophic palatine tonsils but evidenced the presence of a thick discharge on the posterior wall of the pharynx likely originating from the rhinopharynx. The otoscopic exam revealed intact tympanic membranes. Given his medical history and nasal endoscopy, the patient was hospitalized and underwent FESS under general anesthesia.

### 2.2 Surgical procedure

**Fig. 3:**
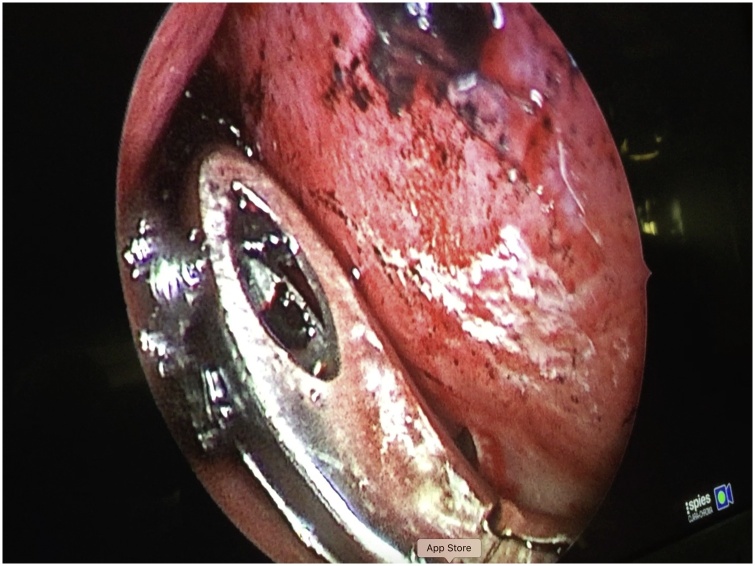
Intraoperative picture. The surgeon retrieves the foreign body from the maxillary sinus.

**Fig. 4:**
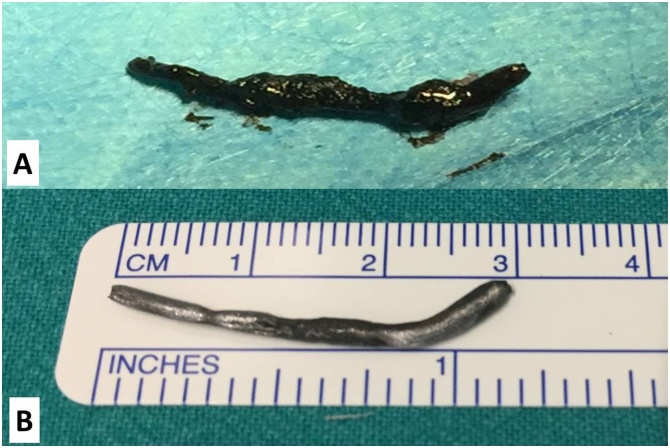
A) The foreign body (metal nail) covered by black encrustations. B) The foreign body after being cleaned.

Firstly, the Otolaryngologist executed uncinectomy and bullectomy. Subsequently, left maxillary antrostomy was performed; after enlarging the ostium of the maxillary sinus, a purulent, blackish and fetid discharge was observed (Fig. 3). The foreign body was located in correspondence to the anterior wall of the maxillary sinus and finally retrieved; it was described as a metal nail covered by black encrustations (Fig. 4 A, B). Final surgical steps consisted of intraoperative control of bleeding and positioning of silastic sheeting in the left middle meatus. In conclusion, the retained foreign body for eight years was the cause of the left maxillary sinusitis.

### 2.3 Post-intervention considerations

No post-operative complications were noted. The patient tolerated the surgical procedure well and was discharged on post-operative day 2.

## 3 Discussion

Unilateral sinusitis is a common pathology. Unlike rhinosinusitis, the inflammatory/infectious disease does not originate from the nose, but exclusively from the sinus. Conversely, the nasal cavities are only secondary affected [13]. USD has a predominant odontogenic origin. Periodontal diseases usually affecting the second upper premolar and molars which are located just next to the maxillary sinus are the most common cause of odontogenic sinusitis [14]. Also, odontogenic sinusitis can occur when part of the restorative dental materials (i.e. metals, porcelains, composite resin) accidentally penetrate into the sinus. Additionally, the maxillary sinus can be impacted by facial traumas and/or hemorrhage causing inflammation and infections, as a result [15,16]. The peculiarity of this case is related to the onset of clinical symptoms. Even though it can seem unusual, work injuries as described in this report are not infrequent. It is an incorrect assumption that garden tools and machinery are not dangerous and people may feel safe enough by using the necessary personal protective equipment [17]. Also, the gardener of this case report was well equipped and protected by his safety helmet with face shield and earmuffs. Moreover, he was using a certified grass trimmer (Professional Grass Trimmer, model TR 600 by Meccanica Benassi ®-Italy), which was supposed to be safe. However, things did not go as planned. In fact, the grass cutter hooked a nail on the ground and, then, propelled it against the patient’s face, precisely hitting the zygomatic region. The speed and the impact of the nail were surely similar to those of a bullet, so that the object passed below the face shield and penetrated into the skin and the anterior part of the maxillary bone until the ipsilateral sinus. Of course, this is just a speculation of the Authors based on the patient’s history and not supported by a thorough forensic analysis. Although this can represent a limitation of our article, it should be acknowledged that there is only one other published case describing a “retained intranasal ballistic foreign body” due to an air-gun injury and involving an adolescent boy. The foreign body was localized in the left nasal cavity under fluoroscopic guidance and removed with an endoscope [18]. However, ballistic foreign bodies determined by air-gun injuries can more frequently happen in adolescent boys, while the localization in the maxillary sinus and the subclinical presentation experienced by our patient makes the current case report even more unusual and unique.

Another interesting point of discussion is the surgical management of USD and how much this has evolved over the last few years. FESS with maxillary antrostomy allows a single functional approach which safely expose the sinus ostia and restores the ciliary mucous transport. Compared to other surgical techniques such as intraoral and traditional Caldwell-Luc’s (CL) approach, the total FESS to retrieve a foreign body is accompanied by minimal invasiveness and surgical trauma with shorter time to recovery for the patient and consequently lower complication rates [19]. Therefore, we highly recommend a total endoscopic surgical approach whenever possible, or a combined endoscopic and intraoral approach for the most complicated cases [20]. The traditional Caldwell-Luc technique for maxillary sinusitis presents several disadvantages, such as such as large bone removal, numbness of the teeth and flap dehiscence; for all these reasons it should limited to intractable cases and preferably a modified technique should be considered [21]. Additionally, a “wait and see” approach can be considered as well, but only in selected cases or safe foreign body location such as the deep soft tissues. Unfortunately, this is not the case of metallic foreign bodies where associated malignancy has been described [22], as well as ballistic objects, which may potentially damage vital structures (i.e. the orbit, paranasal sinuses, great vessels, cranial nerves, brain etc.) depending on their trajectory and speed [18]. For all these reasons, the conservative management suggested by the first Otolaryngologist was inappropriate, as also evidenced by the patient’s history.

Thanks to this case report, a few other observations can be made. The first one concerns the safety measures currently used. In our opinion, full-face shield helmets represent the best option and should be employed in order to avoid the penetration of any possibly dangerous materials. In fact, if the patient had used a full-face shield helmet, he would have not been injured. Secondly, a comment about grass trimmer designs can be made. In fact, more sophisticated designs are required in order to prevent injuries like this and to throw any objects from the ground (i.e. stones, pieces of glass, metals etc.). From a clinical point of view, the patient presented with a few symptoms initially, so much so that he did not care about the lesion and its potential complications. Moreover, even a small deviation in the object’s trajectory would have led to severe consequences (i.e. ocular lesion with subsequent vision loss, penetration of the foreign body intracranially). The onset of the clinical symptoms due to the presence of the foreign body was worth noting, as well. As observed in the patient’s history, it took eight years to evidence the sinonasal infectious complications caused by the foreign body as well as the smell impairment.

## 4 Conclusions

This case is the further proof that it is important to consider a broad differential in patients with USD and that every patient may have personal features that positively or negatively influences the clinical course and onset of complications.

Although the removal of foreign bodies from the maxillary sinus is a topic well described in the current literature, this case highlights the importance of early diagnosis and treatment. As evidenced by our case, the choice of a functional treatment option had more benefits than a conservative management. In fact, if the first Otolaryngologist who visited the gardener would not have told him not to worry about the presence of the foreign body, the patient would have not experienced the sinonasal inflammatory complications that negatively impacted his psychophysical health and quality of life.

## Declaration of Competing Interest

No conflicts of interest to declare.

## Sources of funding

No source of funding to declare.

## Ethical Approval

This study was conducted in good clinical practice and in accordance with the Declaration of Helsinki.

## Consent

Written informed consent was obtained from the patient for publication of this case report and accompanying images. A copy of the written consent is available for review by the Editor-in-Chief of this journal on request.

## Author contribution

M.G. and N.D. designed the study, participated in data collection and analyses, drafted and approved the final version of this paper. E.M.C.T. participated in data analysis and manuscript preparation, M.C. and N.Q. provided critical comments, and approved the final version of this paper.

## Registration of Research Studies

Name of the registry: N/A

Unique identifying number or registration ID: N/A

Hyperlink to your specific registration (must be publicly accessible and will be checked):

## Guarantor

Eleonora M.C. Trecca, MD

## Provenance and peer review

Not commissioned, externally peer reviewed.
